# A four-marker signature of TNF-RII, TGF-α, TIMP-1 and CRP is prognostic of worse survival in high-risk surgically resected melanoma

**DOI:** 10.1186/1479-5876-12-19

**Published:** 2014-01-23

**Authors:** Ahmad A Tarhini, Yan Lin, Oladapo Yeku, William A LaFramboise, Madeeha Ashraf, Cindy Sander, John M Kirkwood, Sandra Lee

**Affiliations:** 1 University of Pittsburgh Cancer Institute, UPMC Cancer Pavilion, 5150 Centre Avenue, 5th Floor, Suite 555, Pittsburgh, PA 15232, USA; 2 Biostatistics Facility, University of Pittsburgh Cancer Institute, 201 North Craig St., Sterling Plaza, Suite 325, Pittsburgh, PA 15213, USA; 3 UPMC Montefiore/Presbyterian Internal Medicine Residency Program, Department of Medicine, University of Pittsburgh, UPMC Montefiore Hospital, N-715, 200 Lothrop Street, Pittsburgh, PA 15213, USA; 4 University of Pittsburgh Cancer Institute, UPMC Shadyside Hospital, 5230 Centre Avenue, Rm. WG02.11, Pittsburgh, PA 15213, USA; 5 Visiting Scholar, Department of Medicine, University of Pittsburgh Cancer Institute, 5117 Centre Avenue, Pittsburgh, PA 15213, USA; 6 Biostatistics and Computational Biology, Dana-Farber Cancer Institute, 450 Brookline Avenue, Boston, MA 02215, USA; 7 University of Pittsburgh Cancer Institute, 5117 Centre Avenue, Suite 1.32, Pittsburgh, PA 15213, USA

**Keywords:** Melanoma, Adjuvant, TNF-RII, TGF-α, TIMP-1, CRP, E1694

## Abstract

**Background:**

E1694 tested GM2-KLH-QS21 vaccine versus high-dose interferon-α2b (HDI) as adjuvant therapy for operable stage IIB-III melanoma. We tested banked serum specimens from patients in the vaccine arm of E1694 for prognostic biomarkers.

**Methods:**

Aushon Multiplex Platform was used to quantitate baseline serum levels of 115 analytes from 40 patients. Least absolute shrinkage and selection operator proportional hazard regression (Lasso PH) was used to select markers that are most informative for relapse-free survival (RFS) and overall survival (OS). Regular Cox PH models were then fit with the markers selected by the Lasso PH. Survival receiver operating characteristic (ROC) analysis was used to evaluate the ability of the models to predict 1-year RFS and 5-year OS.

**Results:**

Four markers that include Tumor Necrosis Factor alpha Receptor II (TNF-RII), Transforming Growth Factor alpha (TGF-α), Tissue Inhibitor of Metalloproteinases 1 (TIMP-1), and C-reactive protein (CRP) were found to be most informative for the prediction of OS (high levels correlate with worse prognosis). The dichotomized risk score based on the four markers could significantly separate the OS curves (p = 0.0005). When using the four-marker PH model to predict 5-year OS, we achieved an area under the curve (AUC) of 89% (cross validated AUC = 72%). High baseline TNF-RII was also significantly associated with worse RFS. The RFS with high (above median) TNF-RII was significantly lower than low TNF-RII (p = 0.01).

**Conclusions:**

The biomarker signature consisting of TNFR-II, TGF-α, TIMP-1 and CRP is significantly prognostic of survival in patients with high-risk melanoma and warrants further investigation.

## Introduction

The immune system plays a critical role in surveillance, suppression, and ultimately the host inflammatory response to cancer. The fine balance between inflammation and immunosuppression in the tumor microenvironment has been shown to be critical to the balance between ultimate tumor resistance or tumor tolerance, especially in hosts with solid tumors [1]. Recruitment of immune effectors, especially proinflammatory mediators, predicts durable responses and cancer progression free survival [2]. Melanoma is a solid tumor that is well known to elicit a strong immune response and as such, has been the focus of multiple therapies designed to improve the antitumor immune response through vaccines, adoptive transfer of tumor-reactive lymphocytes [3], cytokines and monoclonal antibodies designed to manipulate immune checkpoints [4,5]. The role of vaccination with proteins and peptides has been an area of intense interest [6-8], however many of these studies have been hampered by modest clinical benefits despite initially promising results [7,9,10]. One of the mechanisms involved in immune escape by melanoma cells involves down regulation of the proinflammatory microenvironment by regulatory T cells (T_reg_) via the release of immunosuppressive cytokines such as IL-10 and TGF-β, among several other immune suppressive mechanisms [9,11]. Type II cytokines such as IL-6, TNF-α, IFN-γ and IL-10 have also been shown to be important regulators of melanoma immune tolerance and escape [12]. Recent studies implicate myeloid derived suppressor cells (MDSC) in the induction of CD8+ T cell tolerance in tumor-bearing hosts and that appear to be recruited by tumor-derived soluble factors such as TGF-ß1, IL-10, VEGF, GM-CSF, IL-6 and prostaglandin E2 [13]. Evaluation of such biomarkers in the peripheral blood for their disease prognostic value is particularly desirable, given the accessibility and the ability to perform highly standardized assessments that may have future clinical applications.

High risk melanoma is defined as surgically resectable AJCC stage IIB-III disease comprising primary tumors between 2 and 4 mm in Breslow thickness with ulceration, greater than 4 mm with or without ulceration or primary tumors with associated evidence of regional lymphatic metastases. For stages IIB-IIC, the 10-year mortality rate could be as high as 40-60% and for stage III it ranges from 30-70%, depending on the degree of locoregional involvement [14]. The current standard of care management involves definitive surgical resection followed by adjuvant interferon-α2b (IFN-α) therapy. Studies of high dose IFN-α (HDI) have yielded significant improvements in overall survival (OS) and relapse free survival (RFS) but were also associated with clinically significant adverse events [15]. Identification of significant prognostic markers in this patient population is a critical area of need that may have clinical applications and may guide future adjuvant trials. This may eventually allow us to focus clinical follow up and adjuvant therapy upon those patients with the highest mortality risk sparing lower risk patients from unwanted follow up, treatment toxicity and cost.

The ganglioside GM2-KLH-QS21 (GMK) vaccine is composed of a highly antigenic molecule expressed on melanoma cells (GM2), coupled to an adjuvant (QS21) to promote a robust and lasting inflammatory response [16]. Early studies demonstrated consistent immunogenicity of the ganglioside vaccine GM2 given with Bacillus Calmette-Guérin (BCG) as an adjuvant and a trend towards improved RFS when compared to patients vaccinated with BCG alone. Patients with high titer GM2 antibodies showed increased survival [17]. To increase immunogenicity, GM2 was covalently conjugated with keyhole limpet hemocyanin (KLH) and the saponin adjuvant QS-21 was included, significantly increasing the immunogenicity [8], and supporting the testing of GMK as adjuvant therapy in high risk surgically resected melanoma patients as was performed in the E1694 trial as compared to HDI [18]. This study showed that patients treated with HDI had 33% reduction in relapse risk compared to those receiving the GMK vaccine, with a 28% reduction in the risk of death. Further, another phase III study (EORTC 18961) confirmed the ineffectiveness of GMK vaccine as adjuvant therapy versus observation in high risk AJCC stage II melanoma [19,20]. Taken together, the two studies support the current view of GMK vaccine as a neutral control with no significant impact upon either endpoints of survival or relapse.

In this study nested within the E1694 GMK trial arm, we identify four markers: C-reactive protein (CRP), Tissue inhibitor of Metalloproteinases 1 (TIMP-1), Tumor Necrosis Factor alpha Receptor II (TNF-RII) and Transforming Growth Factor alpha (TGF-α) where the linear combination in the analysis of our model generates a risk score that has a significant prognostic value for high risk melanoma patients. We show that baseline levels of this panel of biomarkers have implications in terms of OS and RFS.

## Methods

### Study design and patients

Banked baseline serum samples from 40 patients participating in the Eastern Cooperative Oncology Group–led intergroup E1694 trial and treated with the GMK vaccine were utilized for this analysis [18]. E1694 was a phase III randomized study of vaccination with GMK (GM2-KLH/QS-21, Progenics, Inc., Tarrytown, NY) versus HDI for resected high-risk cutaneous melanoma patients (defined as T4 > 4 mm primary lesions, or any primary lesion accompanied by regional lymph node metastasis). Patients who were assigned to the vaccine group received GMK vaccination up to 12 times over a 2-year period. All patients had an Institutional Review Board approved written informed consent obtained.

### Procedures

Using standardized phlebotomy procedures, up to 30 ml of peripheral blood was drawn from each of the patients. Samples utilized in this study were obtained from subjects after study enrollment but prior to treatment initiation (baseline). Blood samples were collected without anticoagulant into red top vacutainers and allowed to coagulate for 20-30 minutes at room temperature. Sera were separated by centrifugation, and all specimens were immediately aliquoted, frozen and stored in a dedicated−80°C freezer. No more than 2 freeze-thaw cycles were allowed before testing for each sample [18].

The Aushon Multiplex Platform (Aushon Biosystems, Billerica, MA) was used to simultaneously quantitate the serum levels of 115 candidate analytes. The assay comprises a multiplex sandwich ELISA of monoclonal capture antibodies spotted in custom planar arrays in 96-well micro-titer plates. After serum incubation and washing, a second biotinylated monoclonal antibody to a different site from the capture epitope was introduced and streptavidin-horseradish peroxidase (HRP) was subsequently bound to the biotin site. Luminol Enhancer/Peroxidase solution was added and the HRP catalyzed the oxidation of luminol to 3-aminophthalate resulting in light emission at 428 nm. A chemiluminescent image was acquired and processed using a 4-parameter curve fit program (SearchLight Array Analyst Software) to compare the experimental sample values to a recombinant calibration curve run in parallel wells to derive absolute concentrations adjusted for dilution and quality values.

The analystes tested were human IL-6, IL-7, IL-10, IL-16, TNF alpha trimer, IL-1 beta, IFN alpha, IL-4R, IL-18, RANK-L, IL-1 alpha, IL-2R, IL-6R, MPIF-1, Leptin, MIG, GDNF, MIP-1 alpha, MIP-1 beta, MIP-1 delta, ITAC, GM-CSF, MCP-4, MIP-3 alpha, MIP-3 beta, MMP-1, SP-C, Amphiregulin, RANK, MCP-2, IP-10, OPG, FGF basic, KGF, HGH, GCSF, MMP-8, MMP-13, TGF alpha, TGF beta 1, TGF beta 2, TRAIL, TARC, MDC, Eotaxin-2, Beta Defensin 2, GRO gamma, TPO, HGF Beta, NGF, ErbB2, EGF, E-Selectin, P-Selectin, E-Cadherin, PAPP-A, VEGF-R1, VEGF- R2, PECAM-1, MCP-1, c peptide, CD40L, TSP-2, H-CC4, BDNF, RAGE, sAPPb, Thrombomodulin, ENA-78, GRO alpha, CD30, ICAM-3, MMP-7, CG alpha, IL-1RII, TNF-RII, IGFBP-3, EGFR, Human Resistin, MMP-9, ICAM-1, VCAM-1, PAI-1 total, IgE, MPO, TIMP-1, TIMP-2, PDGF-AA, NGAL, Acrp-30, MIP-4/PARC, SHBG, CD14, Clusterin, CRP, NAP-2, Fibronectin, PDGF-BB, VEGF-D, VEGF, TWEAK, PDGF-AB, M-CSF, SDF-1 beta, OPN, Ang-2, IL-17A, RBP4, alpha-2 macroglobulin, Apo-A1, Von Willebrand Factor, A-SAA, IL-23, Visfatin, Fibrinogen.

### Statistical analysis

Univariate proportional hazard (PH) models were used to assess the association between each marker and OS or RFS. The Benjamini and Hochberg’s method was used to adjust for multiple testing. Markers with detection rate < 70% were dichotomized as detected versus not detected. The least absolute shrinkage and selection operator proportional hazard regression (Lasso PH) was used to select markers that are most informative for RFS and OS [21]. Markers with fitted coefficients ≥ 0.1 were selected. We then fitted the regular PH models using the markers selected by the Lasso PH. The survival receiver operating characteristic (ROC) analysis was used to evaluate the ability of the models to predict 1-year RFS and 5-year OS [22]. Leave-one-out cross validations (LOOCVs) were used to avoid over fitting.

## Results

A panel of four markers that include Tumor Necrosis Factor alpha Receptor II (TNF-RII), Transforming Growth Factor alpha (TGF-α), Tissue Inhibitor of Metalloproteinases 1 (TIMP-1), and C-reactive protein (CRP), at baseline was found to be most informative for OS (high serum levels correlate with worse prognosis). While only TNF-RII was significantly associated with RFS.

### RFS analysis

**Figure 1:**
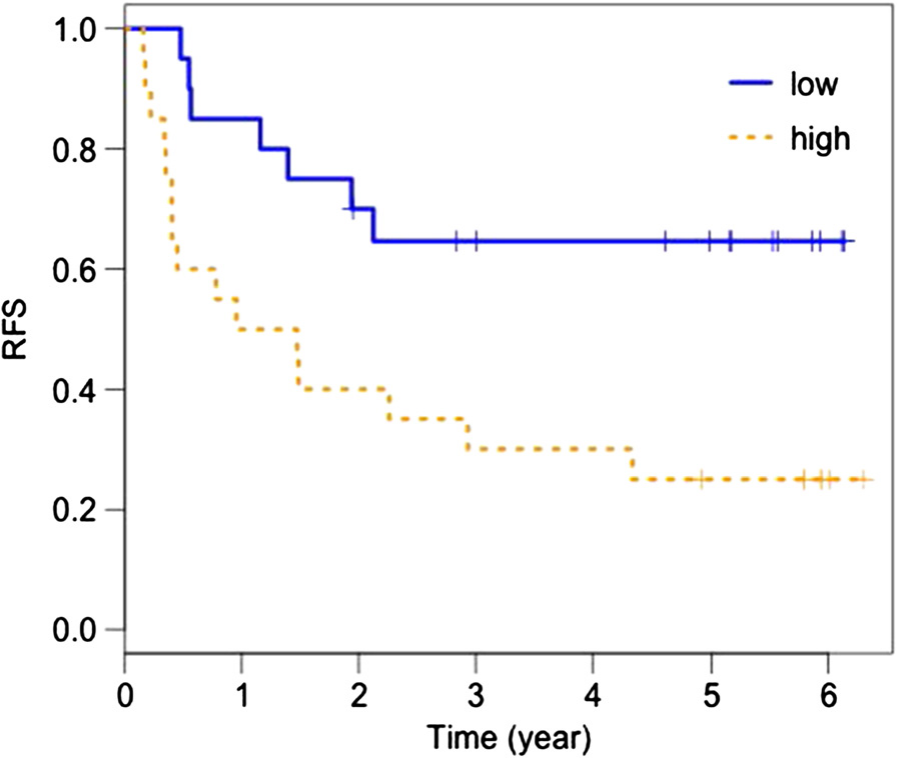
**Kaplan-Meier plot of relapse free survival (RFS) by baseline TNF-RII.** High baseline levels of TNF-RII, dichotomized at the median, are significantly associated with worse RFS (p = 0.01).

TNF-RII was selected as the only main contributor for RFS among the markers tested. We used the Cox PH model with TNF-RII as a predictor for 1 year RFS using survival ROC analysis [22]. We achieved an area under the curve (AUC) of 76% with the full dataset; however, the cross-validated AUC was 66%. High baseline levels of TNF-RII, dichotomized at the median, were significantly associated with worse RFS (log rank test p–value = 0.01). Figure 1 shows the Kaplan-Meier (K-M) plot of RFS by baseline TNF-RII.

### OS analysis

**Figure 2:**
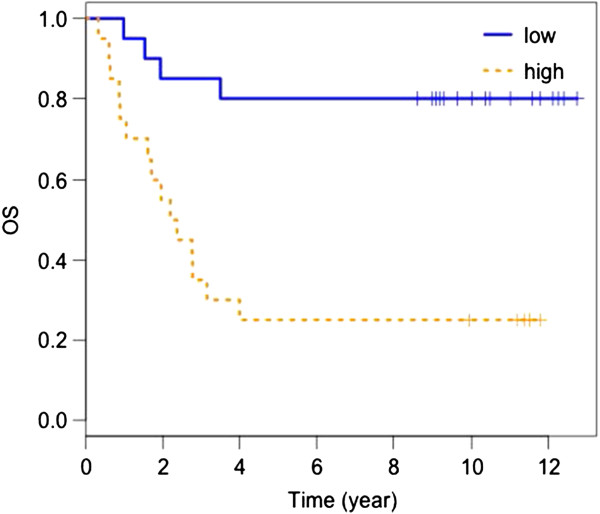
**Kaplan-Meier plot of overall survival by baseline TGF-α, TNF-RII, TIMP-1, CRP (Cox PH model score).** High serum levels correlate with worse prognosis.

Markers with Lasso coefficients >0.1 were included in the final model. The panel of markers that were selected included TGF-α, TNF-RII, TIMP-1 and CRP. The three markers that were most reliable were TNF-RII, TIMP-1 and CRP (being selected by the model most of the time during the cross validation). The survival ROC analysis demonstrated that the Cox PH model using these four markers predicts the 5 year OS well with an AUC for the full dataset of 88% and a cross-validated AUC of 72%. Dichotomizing the linear score by the Cox PH model, at the median, was significantly predictive of OS (log rank test p-value = 0.0005). The K-M plot for the dichotomized score by the Cox PH model is shown in Figure 2.

## Discussion

Among populations of patients with melanoma at high risk after surgical resection, efforts to identify subsets of patients at relatively higher risk for melanoma recurrence and mortality are warranted. These may have clinical prognostic implications, and may drive the design of future adjuvant trials by enabling us to stratify treatment according to risk groups where higher risk patients may be more likely to benefit from adjuvant interventions. Patients treated on the GMK vaccine arm of E1694 are ideal candidates for studies of baseline prognostic biomarkers given the lack of any demonstrable impact this vaccine has shown in terms of either RFS or OS in large randomized controlled multicenter cooperative group trials [6,18,19]. In E1694, the ganglioside GMK vaccine is now therefore considered a control arm of this study, where the clinical outcome showed significant advantage in favor of HDI in an intent-to-treat analysis analysis of both RFS and OS [18]. The GMK vaccine was also evaluated as an arm of the E2696 study which was a randomized phase II trial that enrolled patients with resected stage IIB, stage III, and stage IV disease [6]. Here again, GMK appeared to have no impact on RFS or OS of patients treated on the trial, whether administered as monotherapy or in combination with HDI [6]. Most recently, the EORTC 18961 trial tested the post-operative adjuvant benefit of ganglioside GM2-KLH21 vaccination treatment versus observation in stage II (T3-T4N0M0) melanoma patients, demonstrating the absence of any significant effect of this vaccination upon either of the primary outcomes of RFS or OS [19].

We have therefore conducted a multiplex analysis of 115 candidate serum analytes in patients treated with the GMK vaccine in E1694. Our modeling analysis has shown that the four-marker panel consisting of TNF-RII, TGF-α, TIMP-1, and CRP, at baseline was found to be most informative in regard to OS where high serum levels correlate with worse prognosis. In addition, high baseline TNF-RII was also identified as the most informative marker in relation to worsened RFS. The RFS of patients with high (above median) baseline levels of TNF-RII was significantly lower than that of patients with low (below median) baseline TNF-RII (log rank test p-value = 0.01).

TNF-α is a proinflammatory cytokine that plays a central role in inflammation [23]. It mediates its activities through two cell surface receptors, TNF-RI and TNF-RII that are active in soluble and in membrane-bound forms, and TNF-RII has been shown to have a higher affinity for TNF-α [24]. TNF-RI is constitutively expressed in most tissues while TNF-RII is expressed predominantly on immune cells and endothelial cells [25]. The soluble forms of the receptors have been reported to act as physiological attenuators of TNF-α activity [26], where the shedding of TNF-Rs leads to diminished surface receptors and this process has been proposed to reduce the clinical activity resulting from TNF-α in rheumatoid arthritis [27]. Therefore, it is reasonable to hypothesize that high circulating levels of TNF-RII may attenuate the proinflammatory effects of TNF-α in patients with cancer as part of an overall state of immune tolerance. Further testing of TNF-RII as a potential prognostic marker in patients with high risk melanoma is warranted.

TGF-α is upregulated in several human cancers [28], including melanoma [29]. It is a polypeptide growth-stimulating factor that has been implicated in the progression of gastrointestinal cancers. In addition, serum TGF-α was found to be significantly elevated in patients with gastric, pancreatic, colon, rectal and esophageal cancers as compared with healthy controls [28]. In melanoma, transcriptional upregulation of TGF-α has been associated with differentiation of human melanoma cells [30]. TGF-α belongs to the epidermal growth factor (EGF) family of mitogens [31]. EGF and TGF-α have closely related tertiary structures and they compete for binding to the EGF-receptor which has been shown to be overexpressed in melanoma cells [31]. The potential prognostic value of high serum levels of TGF-α in high risk melanoma patients at poorer prognosis should therefore be further evaluated as supported by our findings.

TIMPs are encoded by four genes designated TIMP-1, TIMP-2, TIMP-3, and TIMP-4 [32]. They are potent regulators of the proteolytic activity of matrix metalloproteinases [33]. TIMP-1 was shown to have cell growth promoting properties in a variety of cancer cell lines including breast carcinoma cells and leukemic cell lines [32]. In addition, TIMP-1 was reported to have anti-apoptotic properties [34], and to have a role as a promotor of cell differentiation [32]. Significantly increased serum TIMP-1 levels and a potential poor prognostic role have been reported in patients with melanoma [35], and patients with a variety of cancer types including breast [36,37], prostate [38], gastric [39], colorectal carcinoma [40], glioblastoma [41], and multiple myeloma [42,43]. In patients with melanoma, levels of TIMP-1 were found to be significantly higher in patients with unresected stage IV disease than in patients with resected stage I/II [35]. In patients with non-small cell lung cancer who underwent surgical resection with curative intent, postoperative TIMP-1 has been reported as an independent predictor of prognosis [44].

Data support a role for high serum CRP as a marker of poor prognosis and of immune tolerance in advanced melanoma [45]. For first detection of melanoma stage IV disease, serum CRP has been reported to be potentially superior to serum LDH measurement [46]. As interesting, is a potential role for CRP in mediating immune tolerance: CRP is synthesized by hepatocytes in response to interleukin-6 during inflammation in concentrations that vary between non-tolerogenic and tolerogenic levels. There is a physiological role of “ectopic” thymic expression in tolerance induction by CRP (and other acute-phase proteins) and possibly other inducible self-antigens [47,48]. CRP binds to phosphocholine (PC) and related molecules on microorganisms and plays an important role in host defense. Further, an important effect may relate to the binding of CRP to PC in damaged membranes. CRP increases clearance of apoptotic cells, binds to nuclear antigens and by masking autoantigens from the immune system or enhancing their clearance, CRP has been hypothesized to prevent autoimmunity [47]. Baseline (pre-treatment) serum CRP was reported to have a potential therapeutic predictive value in melanoma patients treated with the anti-CTLA4 antibody tremelimumab [49,50].

Our systematic modeling analysis starting with the 115 markers tested utilizing baseline biospecimens identified the signature of four markers discussed (TNF-RII, TGF-α, TIMP-1, and CRP) where the linear combination generates a risk score that is significantly prognostic of worse survival in this patient population. The ROC analysis has further supported the survival predictive ability of the model. Further characterization and validation of this association for the individual markers as well as the four markers signature is indicated both in terms of clinical applications as markers of poor prognosis in patients with high risk melanoma after surgical resection and in the design of future adjuvant trials as stratification factors once validated.

## Conclusion

The four serum biomarker signature consisting of TNF-RII, TGF-α, TIMP-1, and CRP as measured at baseline is significantly prognostic of worse survival in high risk melanoma patients and warrants further investigation as a marker of poor prognosis that my guide patient follow up and the design of future adjuvant studies.

## Abbreviations

Treg: Regulatory T cells; MDSC: Myeloid derived suppressor cells; HDI: High dose IFN-α; IFN-α: Interferon-α2b; OS: Overall survival; RFS: Relapse free survival; GMK: Ganglioside GM2-KLH-QS21 vaccine; BCG: Bacillus Calmette-Guérin; KLH: Keyhole limpet hemocyanin; CRP: C-reactive protein; TIMP-1: Tissue inhibitor of metalloproteinases 1; TNF-RII: Tumor necrosis factor alpha receptor II; TGF-α: Transforming growth factor alpha; HRP: Streptavidin-horseradish peroxidase; PH: Proportional hazard; ROC: Receiver operating characteristic; LOOCVs: Leave-one-out cross validations.

## Competing interests

The authors declare that they have no competing interests.

## Authors’ contributions

AAT and JMK were responsible for study design, study conduct, data analysis, and manuscript writing. YL conducted data analysis and contributed to manuscript writing. OY participated in study conduct and in manuscript writing. WAL participated in study conduct and in manuscript writing. MA participated in the study conduct and manuscript writing. SL participated in data analysis and manuscript writing. All authors read and approved the final manuscript.
